# Electricity

**Published:** 1849

**Authors:** A. C. Jackson


### ARTICLE III.

*Electricity.* A paper read before the Union Medical Society of Northern Indiana. By Dr. A. C. Jackson.

From experiments made on animals by Dr. Wilson Phillip in 1822, it was pretty satisfactorily determined that the nervous system constitutes a perfect set of conductors, through which Electricity is conveyed to every part of the body; and that this principle is necessary to give a healthy action to all the secretions and excretions of the system.

In these experiments, often repeated "the eighth pair of nerves, which are chiefly distributed to the stomach and lungs, were divided in the neck of several rabbits shortly after having fed upon parsley. Their respiration was thus immediately rendered laborious; nausea and frequent attempts to vomit supervened; and the animal finally died, apparently of suffocation. Upon opening their stomachs, also, the parseley was found quite unaltered, from which he concluded that the secretions of gastric juice, had been suspended. The same experiment was then performed upon other rabbits, with this difference, that galvanic currents were sent to the stomach by applying one of the poles of a small pile to the lower ends of the divided nerves, and the other to the epigastrium. In all these cases dyspnœa and a tendency to vomit were wanting, and the animals being killed after the current had been continued for 26 hours, the parsley was found perfectly digested, and the stomach exhaled the odor peculiar to this organ during digestion."

From experiments such as these, very frequently repeated, and always with the same results, Dr. Phillip concludes "that the secretion of gastric juice is under the control of the nervous influence; and that this latter is identical with, because it may be replaced by galvanism." And further he says "galvanism is capable of performing all the functions of the nervous influence in the animal economy."

Electricity has been recommended and used as an agent in the cure of some diseases for nearly a hundred years, but why has it not been more generally introduced? From this theory, advocated for the last twenty five years, and from empirical knowledge of its delightful effects in the cure of a great variety of disease, why does not electricity, galvanism, or the more modern electro magnetism, stand out as one of the most important remedial agents of which physicians are possessed, and with which the world is blessed? Surely if its importance was appreciated, instead of seeing the machine or battery in the laboratory of the chemist alone, we would find it in the office of every physician. and at the bedside of every patient.

This agent would appear to be of more importance to us, as physicians, in this climate than in almost any other. A great majority of the cases we are called to treat, are more of a debilitated nervous, than an inflammatory character. There is something in the nature of the climate that tends to depress the power and energy of the system. In our treatment of disease, generally, we resort more to the use of tonics and stimulants than to depletory means. All the uncomplicated periodical diseases we see, are known to be of this character. Whatever theory is adopted with regard to the cause of intermittents or remittents, all agree that the impression is upon the nervous system. Our lingering agues, a thousand and one, our slowly convalescents from a more severe attack of fever; the sallow, bilious complexioned, we see ever day, with a torpid condition of all the secretions, all demand a supporting course. And in connection with our quinine, strychnine, &c., we might do well to use electricity to invigorate the system, and to restore the secretions,

In almost all cases of indigestion there is only a want of nervous influence. Such patients have diverted a due supply of nervous energy from the stomach, by intense mental application, or the stomach has become weakened from sympathy with a deranged system generally. In either case, from the experiments of Dr. Philip, the use of magnetism would be indicated, and its effects, in connection with the hygienic course generally recommended, would surely be the most delightful. For in all such cases, where the nervous system is not supplied with stimulous enough to feed all parts and functions of the body, it would most surely be good practice to substitute a principle of the same power, or, rather, directly add a supply of the same principle.

In some cases of amenorrhœa we have almost the same state of things; an atonic condition of the system generally, with a want of uterine action. In our attempts to restore, we try to give tone and vigor to the system, and determine action to the pelvic viscera. Perhaps with no one means could we do half so much to meet the indications, in such a case, as with electricity.

Paralysis, amaurosis, deafness, &c., are affections in which above all, galvanism most naturally suggests itself. In these it has a long time been used, and has gloriously signalized its worth in giving sight to the blind, and hearing to the deaf; in bidding the dumb to speak, and the palsied body to arise and walk. In the cure of these we make a direct application to the principle, unless there be structural derangement of the nerves, the conductors are there but the flow of electricity they are wont to convey, is not regularly supplied, consequently there is imperfect sensation and motion, imperfect sight, and imperfect hearing. It is an interesting fact, and one that goes strongly to support this theory, that in all these instances electricity supplies the place of nervous influence. In paralytic parts, in which sensation and motion are gone, galvanism is known to excite instantaneous action. When a current of electricity is thrown upon the retina of amaurotic patients, light is seen, though there was darkness before, and objects are said to be discerned clearly, where there was imperfect vision.

Galvanism is said to have magic power in asphyxia, in violent concussion of the brain, and in deadened sensibility of the nerves from narcotic poisons. These are cases liable to occur any day in the practice of any physician. And though he be called to only one in a long life of practice, it is no less his duty and interest to be supplied with the most effectual means to meet the emergency of the case. Here, as in other instances in practice, the life of the unfortunate patient, as well as the hopes of the friends, depends upon the efficiency of the means of him to whom they have confiided the case. And should he be successful in the use of this agent in arousing and reanimating the almost lifeless body, as by magic, it would be well enough for the patient, delight enough for the friends, and glory enough for the physician.

But it is useless for me to specify cases in which this agent would be indicated, and in which it is recommended, as these are more familiar to you all. It is not only applicable in diseases of debility, but also when there is an irritable condition of the nervous system. It may have an exciting or sedative effect, according as it is applied. Dr. Dunglison says, "if a prepared frog be subjected to a quick succession of electric shocks, its muscles are rendered permanently rigid, and it exhibits all over the appearance of a tetanic patient; but now, passing through it for same time, a continuous current in the opposite direction, the muscles gradually relax and finally attain their natural state." And further, he says, "if this action be continued for sometime, the muscles become insensible, and the excitability, in a great measure, is destroyed; for, on subsequently applying shocks, no spasms, or very feeble ones, will be produced." Now this gives us a general direction for the application of electricity or electro magnetism. If we wish to arouse the system, or any function, we place the positive pole near the origin, and pass a succession of shocks in the direction of the ramification of the nerves of the parts we wish to excite. On the contrary, where a sedative effect is wanted, we use the continued current and pass it in the opposite direction.

We have used electro magnetism in a variety of cases the past summer; and, in no instance, without a beneficial effect. It is a powerful agent, and surely, from its interest and importance, should demand more attention from the profession.

